# A Multi-Agent RL Algorithm for Dynamic Task Offloading in D2D-MEC Network with Energy Harvesting †

**DOI:** 10.3390/s24092779

**Published:** 2024-04-26

**Authors:** Xin Mi, Huaiwen He, Hong Shen

**Affiliations:** 1 School of Computer, Zhongshan Institute, University of Electronic Science and Technology of China, Zhognshan 528400, China; 202021080226@std.uestc.edu.cn; 2 Engineering and Technology, Central Queensland University, Brisbane 4000, Australia; hongsh01@gmail.com

**Keywords:** MEC, D2D communication, multi-agent reinforcement learning, energy harvesting, dynamic task offloading

## Abstract

Delay-sensitive task offloading in a device-to-device assisted mobile edge computing (D2D-MEC) system with energy harvesting devices is a critical challenge due to the dynamic load level at edge nodes and the variability in harvested energy. In this paper, we propose a joint dynamic task offloading and CPU frequency control scheme for delay-sensitive tasks in a D2D-MEC system, taking into account the intricacies of multi-slot tasks, characterized by diverse processing speeds and data transmission rates. Our methodology involves meticulous modeling of task arrival and service processes using queuing systems, coupled with the strategic utilization of D2D communication to alleviate edge server load and prevent network congestion effectively. Central to our solution is the formulation of average task delay optimization as a challenging nonlinear integer programming problem, requiring intelligent decision making regarding task offloading for each generated task at active mobile devices and CPU frequency adjustments at discrete time slots. To navigate the intricate landscape of the extensive discrete action space, we design an efficient multi-agent DRL learning algorithm named MAOC, which is based on MAPPO, to minimize the average task delay by dynamically determining task-offloading decisions and CPU frequencies. MAOC operates within a centralized training with decentralized execution (CTDE) framework, empowering individual mobile devices to make decisions autonomously based on their unique system states. Experimental results demonstrate its swift convergence and operational efficiency, and it outperforms other baseline algorithms.

## 1. Introduction

With the rapid development of mobile software services, more and more application services are becoming computation-intensive and delay-sensitive, such as virtual reality, augmented reality, and online games. Therefore, the mobile edge computing (MEC) scheme, which allows mobile devices to offload tasks to an edge server close to the end user and significantly reduces the service response delay, is considered to be a promising paradigm and has attracted the attention of researchers [1,2,3].

However, in a large-scale MEC system with numerous mobile devices, the dynamic and sometimes bursty nature of edge device loads can overwhelm the server, potentially hindering the processing of offloaded computation tasks within the required delay constraints. D2D technology emerges as a pivotal solution within the realm of 5G networks [4,5,6,7]. By leveraging idle end devices’ computing resources through D2D communication links, mobile devices can offload tasks to these available resources [6]. Therefore, effectively harnessing these idle computing resources via D2D links to minimize task delays and collaborate seamlessly with edge servers becomes paramount for optimizing the performance efficiency of MEC systems.

Numerous studies have focused on reducing computation task delays in MEC systems [8,9,10,11,12]. However, most of these works assume that tasks are divisible and can be completed within a single time slot. Tang and Wong [13] introduced a long-term cost minimization algorithm for an MEC system that accounts for non-divisible, delay-sensitive tasks and dynamic edge loads spanning multiple time slots. Nevertheless, their approach did not consider integrating D2D communication or consider the energy harvesting constraints of mobile devices. Reinforcement learning (RL) has emerged as a promising approach to tackle the computational complexities associated with MEC systems [14]. It has been leveraged to develop algorithms for task offloading in dynamic MEC environments [3,10,15]. Li et al. [16] utilized the deep Q-network (DQN) to jointly manage computation offloading and resource allocation in multiuser MEC setups to minimize the total cost of delay and energy consumption. Chen et al. [10] combined residual blocks, long short-term memory, and rank-based prioritized experience replay (rPER) within the deep deterministic policy gradient (DDPG) algorithm to address the joint optimization of computation offloading and resource allocation. In our prior study [5], we introduced an independent PPO-based task-offloading algorithm for D2D-MEC systems with energy harvesting, focusing on task-specific latency constraints. Each agent operates a separate PPO model independently, training on its own state information, lacking information sharing and collaboration, resulting in slow convergence. However, in a large-scale MEC system, RL algorithms relying on a single agent may encounter challenges such as action-space explosion, leading to slow convergence and suboptimal performance [17]. Distributed multi-agent reinforcement learning techniques, which focus on local information and offer easier deployment, align well with the architectural requirements of MEC systems.

In this paper, we investigate the computation-task-offloading problem in a D2D-MEC network under harvested energy constraints. The problem aims to minimize the average task service delay by determining the CPU frequency for each active mobile devices and making task-offloading decisions for each task during each time slot. We utilize a queuing system to represent non-divisible tasks and account for computation tasks that extend over multiple time slots. To address the stochastic nature of task generation, dynamic channel states, and unpredictable harvested energy, we frame the problem of minimizing the average task service delay as a sequential decision challenge, involving a vast multi-dimensional discrete action space. To tackle this complexity, we introduce a multi-agent RL algorithm based on the MAPPO technique, which enables each agent to autonomously make decisions, thereby reducing the decision space. Our proposed algorithm adopts a centralized training approach coupled with decentralized execution, enhancing its practical applicability in real-world scenarios. Experimental results show that our proposed algorithm can work efficiently in a distributed manner without requiring system information prediction.

Our key contributions are summarized as follows:

- We propose a novel scheme that leverages dynamic voltage and frequency scaling (DVFS) and energy harvesting (EH) to enhance energy efficiency and minimize task delays in MEC networks operating under energy constraints. This scheme accounts for stochastic environmental factors such as dynamic harvested energy, fluctuating communication channel conditions, and random task generation. By employing a queuing system to model computation tasks spanning multiple time slots with varying processing speeds and data transmission rates, we accurately assess the edge load of each mobile device in a D2D-MEC network.
- To address the curse of dimensionality inherent in sequential Markov decision processes for solving the nonlinear integer programming (NLP) problem of task offloading, we propose the MAOC algorithm by leveraging the multi-agent proximal policy optimization (MAPPO) technique. Our algorithm adopts a CTDE framework, enabling each mobile device to autonomously make decisions based on its system state. This approach is practical for real-world deployment.
- Through comprehensive simulations, we validate the efficacy of our proposed algorithm. The numerical results demonstrate the algorithm’s rapid convergence and superior performance compared to baseline algorithms.

The remainder of this paper is organized as follows: Section 2 introduces the related work. Section 3 presents our system model. Section 4 provides the mathematical formulation of the problem. Section 5 gives the details of the algorithm design and implementation for the optimization problem. In Section 6, simulations are conducted to verify the performance of our proposed algorithm. The conclusions are finally drawn in Section 7.

## 2. Related Works

MEC stands out as a promising paradigm for 5G heterogeneous networks, attracting significant attention [10,18,19]. Machine learning algorithms play a pivotal role in enabling intelligent decision making for task offloading in MEC systems, adapting to the stochastic and dynamically changing environment. Cao et al. [4] reviewed intelligent offloading in multi-access edge computing, highlighting various research endeavors employing ML-based methodologies. Chen et al. [10] proposed a Temporal Attentional Deterministic Policy Gradient (TADPG) algorithm to minimize the average long-term cost of computational tasks in MEC systems. However, prior works assumed that tasks could be completed within a single time slot without impacting subsequent tasks, neglecting scenarios where tasks span multiple time slots. In contrast, Tang and Wong [13] considered the dynamic load at edge device, the non-divisible and delay-sensitive computation tasks, they proposed a learning algorithm integrating LSTM, dueling DQN, and double DQN to minimize task delay in MEC networks. But none of them accounted for D2D communication intricacies and energy constraints.

D2D technology, a pivotal 5G communication innovation, empowers users to offload tasks to neighboring idle devices, thereby enhancing the overall computational efficiency of the system. Wang et al. [9] delved into the task-offloading conundrum, aiming to minimize the weighted sum of delay and energy consumption across multiple independent subtasks within a D2D-assisted MEC framework. Chai et al. [20] formulated the task execution cost minimization challenge as a mixed-integer nonlinear problem, proposing a heuristic algorithm grounded in the Kuhn–Munkres algorithm and Lagrangian dual method.

DVFS and energy harvesting are two important technologies for saving energy and prolonging the lifetimes of batteries in end devices, and they have caught the attention of researchers in recent years. Liang et al. [21] proposed a joint re-ordering and frequency scaling (JRFS) algorithm to minimize makespan in an MEC network, considering precedence constraints among tasks and using DVFS technology to scale the frequencies of edge servers. Xia et al. [22] investigated an EH-enabled MEC offloading system and proposed an online distributed optimization algorithm based on game theory and perturbed Lyapunov optimization theory. Guo et al. [18] formulated an EH computation-offloading game to minimize delay in an MDC system with energy harvesting and developed a distributed EHCOG scheme to solve it. However, the unpredictable amount of harvested energy in D2D-MEC networks also poses challenges for making collaborative task execution decisions.

To tackle the stochastic and dynamic environment of MEC systems, deep reinforcement learning approaches are used to design algorithms for task offloading in MEC networks. Chen et al. [23] investigated the long-term utility performance maximization problem for an MEC with an ultra-dense sliced radio access network, and proposed two double-DQN-based online strategic computation-offloading algorithms. Huang et al. [24] proposed a DQN-based algorithm for joint task offloading and bandwidth allocation in a multi-user MEC system. Huang et al. [25] proposed a DQN-learning-based online offloading algorithm, DROO, for binary computation of offloading in wireless-powered MEC networks. Huang et al. [15] proposed a task-offloading scheme, RRLO, based on DVFS energy consumption reduction, by using the double Q-learning algorithm. But all the above studies are based on single-agent learning algorithms, which may incur difficulty in convergence and bad performance [17] when used on large numbers of multiple devices due to the curse of dimensionality. [26] proposed a novel DRL algorithm based on the latent space to optimize the trajectory of multiple unmanned aerial vehicles (UAVs), considering the task priorities and binary offloading mode in a UAV-enabled MEC network.

## 3. System Model

**Figure 1:**
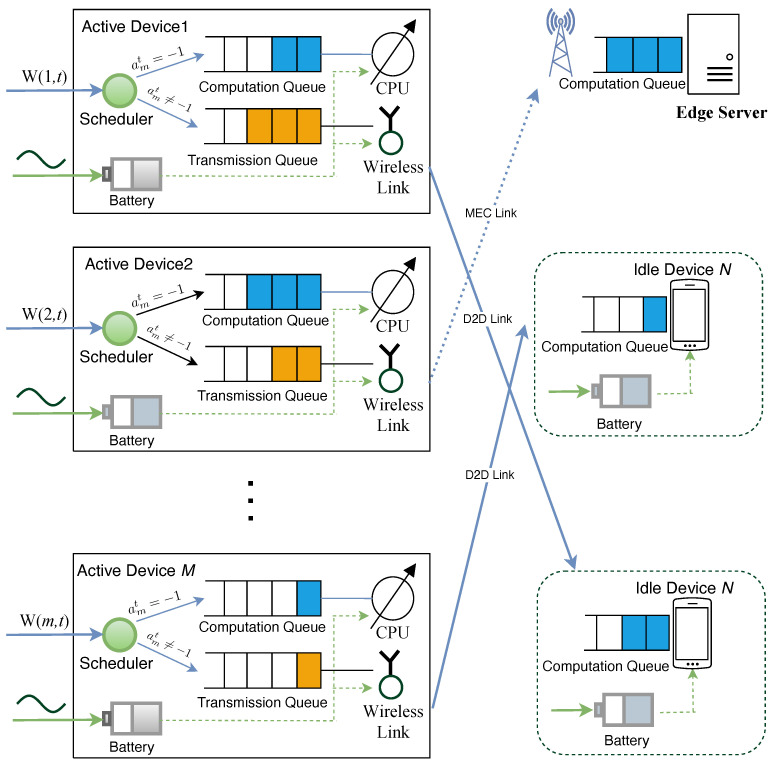
Architecture of D2D-MEC network.

In this paper, we consider a D2D-MEC system comprising an edge server and a collection of MDs, as illustrated in Figure 1. We assume that the system operates in discrete time slots, with the entire period divided into *T* time slots, represented as T={1,2,⋯,T}. Each time slot has a duration of ∆t, which is sufficiently small to ensure that no more than one task is generated by each device within a slot.

In the following subsections, we present the details of the device model, computation model, transmission model, energy model, and delay model employed in the system. The key notation used in this paper is summarized in Table 1.

### 3.1. Device Model

In the D2D-MEC system, the device set D is divided into two subsets: the active device subset $M=\{M_{1},M_{2},\cdots ,M_{\left|M\right|}\}$, and the idle device subset $N=\{N_{0},N_{1},N_{2},\cdots ,N_{\left|N\right|}\}$, as depicted in Figure 1. Here, $N_{0}$ is the edge server and D=M∪N. We assume that idle devices can handle offloading tasks independently without forwarding them to edge servers, thereby alleviating the load on the latter [2,27]. Each MD d∈D employs battery $RB_{d}$ and integrates with the energy-harvesting module to enhance performance. The edge server, on the other hand, is not subject to energy constraints as it is directly connected to base stations via wired connections [27,28,29].

In each time slot *t*, a computation task is generated exclusively on each active device. The task has a specific size and computational complexity, a task arriving at *m* in slot *t* is denoted as $w_{m}^{t}=\left(s_{m}^{t},c_{m}^{t}\right)$, where $s_{m}^{t}$ represents the task size in 1-bit units, and $c_{m}^{t}$ represents the computational complexity measured in CPU cycles required for a 1-bit operation. It should be noted that we assume that task $w_{m}^{t}$ is indivisible and may span across multiple consecutive time slots.

To accurately assess the edge load of each MD, we employ a queuing system to represent the task execution process. Computation tasks are enqueued and scheduled for processing in a first-come, first-served (FCFS) manner. As illustrated in Figure 1, the D2D-MEC system consists of two types of queues: the computation queue $Q_{d}^{comp}$, used for task execution, where d∈D; and the transmission queue $Q_{m}^{tran}$, used for task transmission only on the active mobile devices, where m∈M.

When a task is generated on an active MD *m*, the scheduler determines the location for its execution. Generally, there are three choices: local execution, offloading to an edge server via cellular links, or offloading to idle devices via D2D links. We use $a_{m}^{t}\in \left[-1,0,1,\cdots ,|N|\right]$ to denote the decision on how to execute task $w_{m}^{t}$. If $a_{m}^{t}=-1$, the computation task will enter the local computation queue $Q_{m}^{comp}$; if $a_{m}^{t}=0$, task $w_{m}^{t}$ will be offloaded to the edge server; otherwise it is offloaded to the $a_{m}^{t}$th device in the idle devices set N.

### 3.2. Computation Model

Active devices, m∈M, leverage DVFS technology to regulate their CPU frequency, $f_{m}\in F$, aiming to conserve power given their limited battery capacity. Nevertheless, it is crucial to acknowledge that lowering the CPU frequency can potentially lead to increased task delays, potentially breaching the user’s service-level agreement (SLA). Here, we embrace a discrete CPU frequency model, where $F=\{f^{1},f^{2},\cdots ,f^{K}\}$ signifies the available CPU frequency options for each active device. Notably, we assume that the CPUs of idle MDs, indexed by *n*, where n∈[1,|N|], maintain a fixed frequency of $f_{n}^{idle}$, while the edge server operates at a substantially higher CPU frequency, as $f_{0}^{idle}$.

Let $\tilde{t}$ represent the time slot when task $w_{m}^{t}$ is placed in the computation queue. In the case of local computing, we have $\tilde{t}=t$. Once task $w_{m}^{t}$ enters the computation queue of MD *d*, d∈D, it will be scheduled for processing at the subsequent slot following the completion of preceding tasks [13]. We define $l_{m,d}^{comp}\left(t\right)$ as the time slot when task $w_{m}^{t}$ is completely processed on the designated device *d*. The duration that task $w_{m}^{t}$ spends in the computation queue can be expressed as the difference between the time of complete processing and the time of entry into the queue:

(1) $$\phi_{m,d}^{comp}\left(t\right)={\left[\max_{t^{\prime }\in \left\{0,1,\cdots ,\tilde{t}-1\right\},d^{\prime }\in \left[1,|M|\right]}l_{d^{\prime },d}^{comp}\left(t^{\prime }\right)-\tilde{t}+1\right]}^{+}$$

where the operator ${\left[x\right]}^{+}=\max\left\{0,x\right\}$, and $l_{d^{\prime },d}^{comp}\left(0\right)$ is set as 0 for simplicity of presentation. The term $\max_{t^{\prime }\in \left\{0,1,\cdots ,\tilde{t}-1\right\},d^{\prime }\in \left[1,|M|\right]}l_{d^{\prime },d}^{comp}\left(t^{\prime }\right)$ represents the time slot when all the task placed in the computation queue before time slot $\tilde{t}$ has been processed. Hence, $\phi_{m,d}^{comp}\left(t\right)$ determines the number of waiting time slots in the computation queue for task $w_{m}^{t}$.

Let $\hat{t}=\tilde{t}+\phi_{m,d}^{comp}\left(t\right)$ denote the time slot in which task $w_{m}^{t}$ starts to be processed at MD *d*. Therefore, we can conclude that task $w_{m}^{t}$ will have been processed completely at time slot $l_{m,d}^{comp}\left(t\right)$, as

(2) $$l_{m,d}^{comp}\left(t\right)=\tilde{t}+\phi_{m,d}^{comp}\left(t\right)+\underset{\theta }{\mathrm{argmin}}\left\{\sum_{i=\hat{t}}^{\hat{t}+\theta }f_{d}\left(i\right)∆T\geq s_{m}^{t}c_{m}^{t}\right\}-1$$

where ⌈·⌉ is the ceiling function. $f_{d}\left(i\right)$ is the CPU frequency of MD *d* in time *i*. In particular, the term $\underset{\theta }{\mathrm{argmin}}\left\{\sum_{i=\hat{t}}^{\hat{t}+\theta }f_{d}\left(i\right)∆T\geq s_{m}^{t}c_{m}^{t}\right\}$ calculates the minimum value of θ such that the total number of time slots required to fully process the task is satisfied.

Here, Equation (2) can be simplified under the following conditions:

(1) For local execution. We have $t=\tilde{t}$, so the complete time slot of task $w_{m}^{t}$ can be written as

(3) $$l_{m,m}^{comp}\left(t\right)=t+\phi_{m,m}^{comp}\left(t\right)+\underset{\theta }{\mathrm{argmin}}\left\{\sum_{i=t}^{i+\theta }f_{d}\left(i\right)∆T\geq s_{m}^{t}c_{m}^{t}\right\}-1$$

(2) For remote execution, considering that the CPU frequency is fixed for idle MDs throughout the entire time period, we have

(4) $$l_{m,d}^{comp}\left(t\right)=\tilde{t}+\phi_{m,d}^{comp}\left(t\right)+\left\lceil \frac{s_{m}^{t}c_{m}^{t}}{f_{d}∆T}\right\rceil -1,\forall d\in N$$

In scenarios where multiple offloaded task from different sources arrive at the target device simultaneously, they will be enqueued in the computation queue based on the following rule: among the offloaded tasks, tasks with lower computing demand, such as $s_{m}^{t}c_{m}^{t}$, will be prioritized and placed ahead in the queue.

### 3.3. Transmission Model

Each active MD m∈M maintains a transmission queue $Q_{m}^{tran}$ to handle task offloading to remote devices. When a task $w_{m}^{t}$ is selected for remote execution, it will be enqueued in the transmission queue and will start to transfer at the next time slot when the previous task has been successfully transferred.

Considering a rapidly varying communication environment, the data transmission rate of mobile device *m* can be determined based on Shannon’s theorem as follows:

(5) $$r_{m}\left(t\right)=B_{m}\left(t\right)\log\left(1+\frac{p_{m}h_{m}^{d}\left(t\right)}{N_{0}}\right)$$

where $B_{m}$ represents the bandwidth allocated to MD *m* and $p_{m}$ denotes transmission power of MD *m*, both of which are constants, as in [22,30]. Here, we assume that cellular links and D2D links operate on different frequency bands and adopt orthogonal frequency-division multiple access (OFDMA) for access. Therefore, communication between any two devices does not interfere with the communication between other devices [20]. In practical settings, the bandwidth available on edge servers significantly exceeds that allocated for D2D communication with mobile devices. While we have not rigorously distinguished between the bandwidth assigned for cellular network communication and D2D communication for simplicity, it is important to note this distinction. However, our algorithm is easily adaptable to incorporate and optimize for this differentiation. $h_{m}^{d}\left(t\right)$ represents the channel gain between MD *m* and target device *d* at time *t*, which remains fixed within a time slot and follows a Rayleigh distribution that varies over time. Additionally, $N_{0}$ represents the white noise of the channel. Therefore, similar to the computation model, we can obtain the number of waiting slots for task $w_{m}^{t}$ in the transmission queue as follows:

(6) $$\phi_{m}^{tran}\left(t\right)={\left[\max_{t^{\prime }\in \left\{0,1,\cdots ,t-1\right\}}l_{m}^{tran}\left(t^{\prime }\right)-t+1\right]}^{+}$$

where $\phi_{m}^{tran}\left(0\right)$ is set to be 0 for presentation simplicity. The term $\max_{t^{\prime }\in \left\{0,1,\cdots ,t-1\right\}}l_{m}^{tran}\left(t^{\prime }\right)$ represents the time slot when all tasks in the transmission queue before *t* have been scheduled.

Let $\overset{ˇ}{t}=t+\phi_{m}^{tran}\left(t\right)$ represent the time slot for starting to transmit task $w_{m}^{t}$. Therefore, for task $w_{m}^{t}$ generated in MD m∈M, it will be completely transmitted by time slot $l_{m}^{tran}\left(t\right)$, which can be expressed as

(7) $$\begin{array}{c} l_{m}^{tran}\left(t\right)=t+\phi_{m}^{tran}\left(t\right)+\underset{\theta }{\mathrm{argmin}}\left\{\sum_{i=\overset{ˇ}{t}}^{\overset{ˇ}{t}+\theta }r_{m}\left(i\right)∆T\geq s_{m}^{t}\right\}-1 \end{array}$$

where $r_{m}\left(i\right)∆t$ denotes the total transfer data size in time slot *i*, and the term $\underset{\theta }{\mathrm{argmin}}\left\{\sum_{i=\overset{ˇ}{t}}^{\overset{ˇ}{t}+\theta }r_{m}\left(i\right)∆T\geq s_{m}^{t}\right\}$ represents the total time slots required to transfer the data of $w_{m}^{t}$ successfully.

### 3.4. Energy Model

Each mobile device employs an EH module to obtain energy such as wind, solar, and ambient RF. The EH evolves based on i.i.d. and the process is modeled as a random process across the whole time period.

Let $e_{d}^{h}\left(t\right)$ denote the amount of harvested energy at the MD *d* at time *t*, which is intermittent and hard to predict. The harvested energy is stored in the battery to support the running of device. Here, we ignore the energy loss caused by battery charging and discharging for simplicity and focus on energy consumption of local computation and data transmission [31]. We denote the energy level of the battery in MD *d* at *t* as $RB_{d}\left(t\right)\in \left[RB_{d}^{min},RB_{d}^{max}\right]$, where $RB_{d}^{min}$ is the minimum energy required to support the basic functioning of the mobile device system, and $RB_{d}^{max}$ is the maximum capacity of the battery. Therefore, the battery status evolves according to the following equation [28]:

(8) $$\begin{array}{c} RB_{d}\left(t+1\right)=\min\left\{\max\left\{RB_{d}\left(t\right)-e_{d}^{c}\left(t\right),RB_{d}^{min}\right\}+e_{d}^{h}\left(t\right),RB_{d}^{max}\right\} \end{array}$$

where $e_{d}^{c}\left(t\right)$ denotes the energy consumption in time slot *t*, which is computed as

(9) $$e_{d}^{c}\left(t\right)=\left\{\begin{array}{c} 1_{\left(Q_{d}^{comp}\neq \emptyset \right)}\delta f_{d}{\left(t\right)}^{3}∆T+1_{\left(Q_{d}^{tran}\neq \emptyset \right)}p_{d}∆T,\forall d\in M\\ 1_{\left(Q_{d}^{comp}\neq \emptyset \right)}\delta f_{d}^{3}∆T,\forall d\in N \end{array}\right.$$

where 1(·) is an indicator function that outputs 1 when · is true and 0 otherwise, and δ>0 is a coefficient related to the CPU chip architecture. If the computation queue $Q_{d}^{comp}$ is empty, there is no energy consumption for computation. The same is true for transmission queue $Q_{d}^{tran}$. Similar to [22], we assume that if there is not enough energy to support task computation or transmission, the MD system is switched to sleep and blocks the task in queues until there is enough energy in the battery.

### 3.5. Delay Model

For task $w_{m}^{t}$ generated in MD *m*, if $a_{m}^{t}=-1$, which means task $w_{m}^{t}$ will be processed locally, then based on Equation (3) we obtain that the delay in task $w_{m}^{t}$ is $\left(l_{m,m}^{comp}\left(t\right)-t+1\right)$, which can be rewritten as

(10) $$L_{m}^{loc}\left(t\right)=\phi_{m,m}^{comp}\left(t\right)+\underset{\theta }{\mathrm{argmin}}\left\{\sum_{i=t}^{i+\theta }f_{m}\left(i\right)∆T\geq s_{m}^{t}c_{m}^{t}\right\}$$

If $a_{m}^{t}\neq -1$, the task will be processed remotely, according to Equation (7), we have the transmission delays $L_{m}^{tran}\left(t\right)=l_{m}^{tran}\left(t\right)-t+1$, that is, $\tilde{t}=L_{m}^{tran}\left(t\right)+t$. According to Equation (4), we have the processing delay $L_{m,d}^{comp}\left(t\right)=\phi_{m,d}^{comp}\left(t\right)+\left\lceil \frac{s_{m}^{t}c_{m}^{t}}{f_{d}∆T}\right\rceil$. Hence, we have the total delay of remote execution task as

(11) $$L_{m}^{rem}\left(t\right)=L_{m}^{tran}\left(t\right)+L_{m}^{comp}\left(t\right)$$

Therefore, the total delay of task $w_{m}^{t}$ can be derived as follows:

(12) $$L\left(m,t\right)=1_{\left(a_{m}^{t}=-1\right)}L_{m}^{local}\left(t\right)+1_{\left(a_{m}^{t}\neq -1\right)}L_{m}^{rem}\left(t\right)$$

## 4. Problem Formulation

In this paper, we aim to minimize the time-averaged task delay under energy constraints of mobile devices in a D2D-MEC system. At each time slot *t*, the MEC system makes task-offloading decisions $A\left(t\right)=\{a_{1}\left(t\right),a_{2}\left(t\right),\cdots ,a_{\left|M\right|}\left(t\right)\}$ for each generated task and CPU frequency decisions $F\left(t\right)=\{f_{1}\left(t\right),f_{2}\left(t\right),\cdots ,f_{\left|M\right|}\left(t\right)\}$ at each active device to optimize the long-term average task delay without knowing the future information. Decisions $a_{m}\left(t\right)$ and $f_{m}\left(t\right)$ are both discrete variable. Here, we adopt discrete CPU frequencies to represent the set of CPU speeds of the active devices, which are denoted as $F=\{f^{1},f^{2},\cdots ,f^{K}\}$, where $f_{m}\left(t\right)=k$ means the frequency of device *m* is set to $f^{k}$.

For simplicity, we use A, F to represent A(t) and F(t) individually in the following sections. Thus, the time-averaged task delay minimization problem can be formulated as problem P1.

(13a) $$\begin{array}{cc} \; & P1:\;\underset{A,F}{\mathrm{minimize}}\;\frac{1}{T}\sum_{t=1}^{T}\frac{1}{M}\sum_{d=1}^{M}L\left(m,t\right)\\ s.t. \end{array}$$

(13b) $$\begin{array}{cc} \; & \left(2),(4),(5),(6),(7),(8\right) \end{array}$$

(13c) $$\begin{array}{cc} \; & a_{m}\left(t\right)\in \left\{-1,0,1,2,\cdots ,|N|\right\},\forall m\in M \end{array}$$

(13d) $$\begin{array}{cc} \; & f_{m}\left(t\right)\in \left\{1,2,\cdots ,K\right\},\forall m\in M \end{array}$$

(13e) $$\begin{array}{cc} \; & RB_{d}^{min}\leq RB_{d}\left(t\right)-e_{d}^{c}+e_{d}^{h}\leq RB_{d}^{max},\forall d\in D \end{array}$$

Equations (13c) and (13d) define the domain of decisions $a_{m}\left(t\right)$ and $f_{m}\left(t\right)$, respectively. Equation (13e) is battery energy constraint for all mobile devices.

Problem P1 is a nonlinear integer programming (NLP) problem, despite having prior knowledge of all the offline environment information (generated task information, harvested energy, and channel state). Generally, P1 is non-convex and NP-hard, which makes it extremely challenging to solve due to the following reasons: (1) The task generation, the amount of energy harvested, and the communication channel state are fast-varying across time slots, making it difficult to predict accurately, which poses significant challenges in designing an algorithm that can operate online; (2) the varying computing speed and transfer speed in constraints (1) and (4) create a tight coupling, requiring careful decision making for task-offloading and frequency decisions across time slots; (3) the combination decisions A,F grow exponentially with increasing network size, specifically, the whole action space at slot *t* is ${\left(\left(N+2\right)K\right)}^{M}$. Therefore, our objective is seek to design an algorithm based on a DRL technique that can learn from historical environmental data and make rapid decisions.

## 5. Solution with Multi-Agent DRL-Based Algorithm

Proximal policy optimization (PPO) [32] is a policy gradient algorithm based on the actor–critic (AC) framework [33] that has been shown to achieve state-of-the-art performance in a wide range of tasks, and has become a popular choice for many RL applications due to its simplicity and effectiveness. To improve the stability and sample efficiency of traditional policy gradient methods, PPO clips the update step of the policy network to prevent it from changing too much at once. Specifically, PPO uses a surrogate objective function to measure the difference between the updated policy and the previous policy. Thus, here we seek to derive an RL algorithm based on the PPO method and an AC framework.

However, single-agent DRL methods like PPO, which rely on a trial-and-error process to interact with the environment, cannot be directly applied to solve P1. This is because the action space ${\left(\left(N+2\right)K\right)}^{M}$ of P1 grows exponentially, resulting in the curse of dimensionality. Additionally, using single-agent DRL means that the MEC system makes decisions centrally, which introduces a heavy communication burden. To address the challenges, we propose a multi-agent DRL-based dynamic offloading algorithm (MADOA) that builds upon MAPPO, a state-of-the-art multi-agent extension of the PPO method. Our algorithm employs the CTDE architecture, composed of a central controller in the edge server and an intelligence agent in each active MD. Each agent interacts independently with the environment and makes decision individually. The central controller collects the global system state to train the shared critic network, which it uses to evaluate the action decision of each agent. Firstly, we formulate P1 as a cooperative Markov game.

### 5.1. Markov Decision Process of P1

*(1) State space:* In each time slot *t*, agent m∈M observes its local system state. This state includes the generated task information ($s_{m}^{t},c_{m}^{t}$), the amount of harvested energy $e_{m}^{h}\left(t\right)$ and the current battery energy level $RB_{m}\left(t\right)$, the backlog of computation queue $Q_{m}^{comp}\left(t\right)$, the backlog of transmission queue $Q_{m}^{tran}\left(t\right)$, and the network channel state $h_{m}\left(t\right)$. Hence, the local state of agent *m* can be denoted as

(14) $$\begin{array}{c} S_{m}\left(t\right)=\left(s_{m}^{t},c_{m}^{t},h_{m}\left(t\right),RB_{m}\left(t\right),e_{m}^{h}\left(t\right),Q_{m}^{comp}\left(t\right),Q_{m}^{tran}\left(t\right),\mathrm{ID}\left(t\right)\right) \end{array}$$

where $h_{m}\left(t\right)=\left\{h_{m}^{1}\left(t\right),\ldots ,h_{m}^{\left|N\right|}\left(t\right)\right\}$, $Q_{m}^{comp}\left(t\right)=\max_{t^{\prime }\in 0,1,\cdots ,t-1}l_{m}^{comp}\left(t^{\prime }\right)$ represents the maximum waiting time in the computation queue $Q_{m}^{comp}$ (e.g., the backlog of queue at time *t*), and $Q_{m}^{tran}\left(t\right)=\max_{t^{\prime }\in 0,1,\cdots ,t-1}l_{m}^{tran}\left(t^{\prime }\right)$ represents the backlog of the transmission queue at *t*. Specially, the matrix ID(t)=3×|N| represents the energy level, the harvested energy, and the backlog of the computation queue of idle devices set N. The state information of the idle device *n* in ID(t) is denoted as $\left\{RB_{n}\left(t\right),e_{n}^{h}\left(t\right),Q_{n}^{comp}\left(t\right),n\in N\right\}$, where $Q_{n}^{comp}\left(t\right)=\max_{t^{\prime }\in \left\{0,1,\cdots ,t-1\right\},d^{\prime }\in \left[1,|M|\right]}l_{d^{\prime },d}^{tran}\left(t^{\prime }\right)$ is the backlog of the computation queue in device *n*. Note that there is no limit to the energy level and energy harvested in the edge server, so we set them to be −1. We assume that the edge server will collect the state information of idle MDs and broadcast them to active MDs at the end of each time slot.

The global state space S is the Cartesian product of all local states of each active MD, denoted as $\prod_{m}S_{m}$. Since the edge server collects all idle mobile device data at the end of each time slot, the global system state at *t* can be expressed as follows:

(15) $$\begin{array}{cc} \; & S\left(t\right)=\left[S_{1}\left(t\right),S_{2}\left(t\right),\cdots ,S_{\left|M\right|}\left(t\right)\right]=\left(\mathrm{AD}\left(t\right),\mathrm{ID}\left(t\right)\right) \end{array}$$

where AD(t)=7×|M| is the state information of the active MDs set, where row *d* is $\left(s_{m}^{t},c_{m}^{t},h_{m}\left(t\right),RB_{m}\left(t\right),e_{m}^{h}\left(t\right),Q_{m}^{comp}\left(t\right),Q_{m}^{tran}\left(t\right)\right)$. Without loss of generality, if no task is generated at active device *m* in time *t*, the task size $s_{m}^{t}$ and task complexity $c_{m}^{t}$ are all set to be 0.

*(2) Action space:* The local action decisions of agent *m* at time *t* are denoted as $\left(a_{m}\left(t\right),f_{m}\left(t\right)\right)$. These decisions include the task-offloading decision for the current generated task and the CPU frequency selection for the active MD. Therefore, the global action decisions at slot *t* can be represented as (A(t),F(t)).

*(3) Reward:* As a fully cooperative multi-agent learning model, each agent shares a common goal. We use the average task completion delay at time slot *t* as the reward for all agents, which is calculated as follows:

(16) $$r\left(t\right)=\frac{1}{M}\sum_{d=1}^{M}L\left(m,t\right)$$

Since we are considering joint actions among devices, this reward value is used to update the actor network of all devices and the global critic network.

### 5.2. MAOC Algorithm

To tackle the huge action space of P1, a multi-agent DRL technique is utilized to decompose the joint actions of all active devices into independent actions of each active MD. We formulate the problem P1 as a fully cooperative multi-agent RL model, where agents learn to interact with each other and their environment to maximize a shared reward signal.

**Figure 2:**
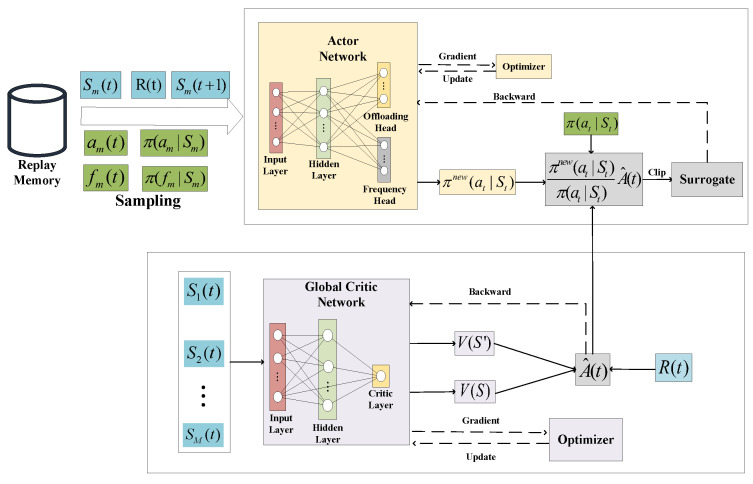
Architecture of the MAOC algorithm.

Utilizing the MAPPO technique, we assign an independent actor network to each device for making action decisions based on local state information, as depicted in Figure 2. Additionally, a global critic network evaluates joint actions using global state information, guiding the actor network updates. Moreover, we implement our MARL algorithm using the CTDE framework, which incorporates a centralized critic for training and multiple decentralized actors that leverage shared experience. This setup allows each agent to make decisions based on its local observations. Our algorithm, named the Multi-Agent Online Control (MAOC) algorithm, consists of two phases: centralized training and decentralized execution.

#### 5.2.1. Centralized Training

The network update mechanism of our proposed algorithm resembles that of the PPO algorithm within the actor–critic framework. To enhance sample efficiency, we incorporate importance sampling to enable the reuse of samples. In order to prevent network instability or crashes caused by aggressive network updates, we employ a policy clip operation to constrain updates within a limited range, ensuring the stability and robustness of the network.

The training phase is illustrated in Figure 2, with the following specific detail: All devices randomly select the same batch of trajectories $\left(S_{m}\left(t\right),a_{m}\left(t\right),\pi \left(a_{m}|S_{m}\right),f_{m}\left(t\right),\pi \left(f_{m}|S_{m}\right),r\left(t\right),S_{m}\left(t+1\right)\right)$ from the experience replay memory.

The communication process among devices during the training phase is as follows: (1) At the end of each time slot, idle MDs send their system state information to the edge server. Upon receiving the information from idle devices, the edge server integrates this data with its own state information and broadcasts it to all active MDs. (2) Each active MD, upon receiving this information, combines it with its own state data to form its system state. At slot t, each active MD interacts with the environment, receives current observations $o_{t}$, and makes stochastic decisions based on its policy. (3) In the next time step, each intelligent agent can observe the state $o_{t+1}$ and the reward $r_{m}\left(t\right)$, and then, send  $o_{t}$, $o_{t+1}$, and $r_{m}\left(t\right)$ to the central controller located at the edge server. (4) Utilizing the acquired data, the edge server makes predictions using the critic network, and calculates the temporal difference (TD) target and TD error. Subsequently, the TD error is broadcast to all active MDs’ intelligent agents by the edge server, which also updates the parameters of the value network. (5) Upon receiving the TD error, each intelligent agent of the active MD updates its policy network.

For the global critic network, the advantage function is calculated using the reward r(t), and then, the network parameters are updated as follows:

(17) $$L_{t}\left(\theta^{critic}\right)=r\left(t\right)+\gamma V\left(S\left(t\right)\right)-V\left(S\left(t+1\right)\right)$$

For the actor network, we use the local state information of the device obtained from the replay memory $S_{m}\left(t\right)$ as input to calculate the current action policy output $\pi^{new}\left(a_{t}|S_{t}\right)$ by the network. We then use importance sampling to calculate the advantage value based on Equation (18) and perform clipping to ensure that the update magnitude is small enough. Finally, we backpropagate and update the actor network parameters.

(18) $$L_{t}\left(\theta^{actor}\right)=\min\left(r_{t}\left(\theta^{actor}\right)\hat{A_{t}},clip\left(r_{t}\left(\theta^{actor}\right),1-\epsilon ,1+\epsilon \right)\hat{A_{t}}\right)$$

where $r_{t}\left(\theta^{actor}\right)=\frac{\pi^{new}\left(a_{t}|s_{t}\right)}{\pi (a_{t}|s_{t})}$ denotes the probability ratio, and ϵ is a hyperparameter that represents the trust region size.

In the loss function of the actor network, the first item refers to conservative policy iteration [34], which can result in an excessively large policy update. The second item modifies the surrogate objective by clipping the probability ratio, which removes the incentive to move $r_{t}$ outside of the interval [1−ϵ,1+ϵ] [32]. By using the minimization operation, the final objective serves as a lower bound on the unclipped objective. As a result, we make a small and safe update, where we only slightly increase the probability of a good action.

Note that we use the generalized advantage estimation (GAE) trick to compute the advantage function, which is as follows:

(19) $${\hat{A}}_{t}=\sum_{l=0}^{\infty }{\left(\gamma \lambda \right)}^{l}\delta_{t+l}$$

where γ is the discount factor, which determines the importance given to future rewards, while λ is a parameter similar to TD(λ), with a trade-off between variance and bias. $\delta_{t+l}^{V}$ refers to the TD error, which is computed as $\delta_{t+l}=r\left(t+l\right)+\gamma V\left(t+l+1\right)-V\left(t+l\right)$, where *V* denotes the critic network operation.

Our proposed centralized training algorithm is summarized in Algorithm 1.
**Algorithm 1:** Multi-Agent Online Control algorithm (MAOC)
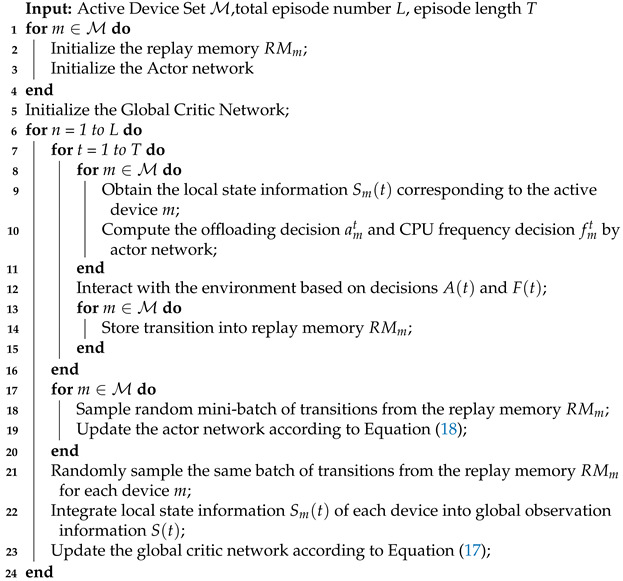


#### 5.2.2. Decentralized Execution

At the end of each time slot *t*, idle devices transmit their own state information to the edge server. Then, the edge server broadcasts this information to active devices, paving the way for the next time slot. Thus, each active device can seamlessly integrate the broadcast information from the edge server and its own current state information into its local state. This local state is then fed into the actor network for inference to obtain the corresponding action policy $\pi (a_{m}|S_{m})$ and $\pi (f_{m}|S_{m})$. The action $a_{m}$ and $f_{m}$ are then obtained through sampling and used to interact with the environment.

#### 5.2.3. Complexity Analysis of MAOC Algorithm

The complexity of the MAOC algorithm is a blend of the initialization and training phases.

During initialization, each active device establishes its replay memory and actor network, with complexity dependent on the number of active devices in set |M|. This phase maintains a constant complexity level as it sets up these components.

In the training phase, iterative episodes unfold where devices engage with the environment, update their actor networks, and contribute to a global critic network. The algorithm complexity is analyzed from the following two aspects. (1) Time step operations: At each time step, tasks such as gathering local state information, decision making with the actor network, environment interaction, and transition storage in the replay memory occur. The complexity per time step is typically linear, influenced by factors like the number of active devices and state space size. (2) Network updates: Updating the actor networks and the global critic network involves sampling from the replay memory, calculating losses, and updating network parameters. The complexity of network updates is related to the size of the replay memory, network architecture, and the number of devices contributing to the global critic network.

## 6. Simulation Results

We construct the reinforcement learning model using the PyTorch framework and train it on a computing server with four GeForce RTX 2080 Ti GPUs and one Intel(R) Xeon(R) Silver 4116 CPU @ 2.10 GHz CPU with 48 cores. After the training, we evaluate the performance of the proposed algorithm through simulation experiments. The environment parameter settings are shown in Table 2 [13,35].

The neural network parameters are as follows: the batch size is set to 64, learning rate is set to 1 × $10^{-4}$, the number of active devices is fixed at 3, the number of idle devices is fixed at 3, and the probability of task generation is fixed at 0.5. The actor network adopts a three-layer multilayer perceptron, while the critic network adopts a four-layer multilayer perceptron. Both networks have a middle layer with 128 nodes, and the Adam optimizer is used to update the networks. We generate training data online using simulators, while test data are pre-generated by simulators and saved to eliminate the influence of simulators on the experimental results.

To evaluate our proposed algorithm, we compare it with the following baseline algorithms:

(1) No D2D mode: This scenario only considers offloading tasks to the edge server and executing tasks locally, without utilizing D2D technology.

(2) All local mode: In this mode, all tasks are executed only on the devices where they are generated, without any task offloading.

(3) PPO algorithm: This approach leverages a single-agent PPO algorithm in the edge server, which collects the state information of all MDs as the training network input. The actor network directly outputs joint actions and broadcasts them to the MDs.

(4) Centralized MAPPO: In this setup, all agents exchange their local state information during the execution phase. Each agent then uses the global state information as input for the actor network to make corresponding decision actions.

**Figure 3:**
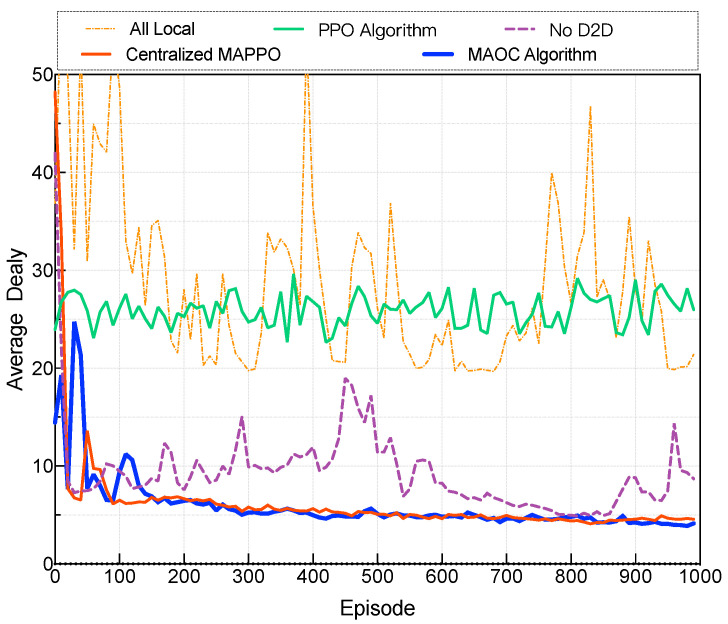
Performance compared with baseline algorithms.

Figure 3 illustrates that our proposed MACO algorithm outperforms the other baseline algorithms. It can be observed that our proposed algorithm demonstrates convergence at around 150 episodes, indicating its effectiveness and feasibility. While our proposed algorithm’s convergence speed may be marginally slower than that of the CM algorithm, its advantage lies in not requiring inter-agent communication during execution. This characteristic reduces data transmission overhead, enhancing its practicality for real-world scenarios. Compared to alternative algorithms, our proposed approach exhibits significant enhancements: an 81.2% improvement compared to all local mode, enhancement compared to PPO algorithms, and a 48.2% boost over the No D2D mode. This underscores the exceptional performance of our algorithm, emphasizing that more input information is not always better. In some cases, redundant information can have a detrimental effect on results [36].

**Figure 4:**
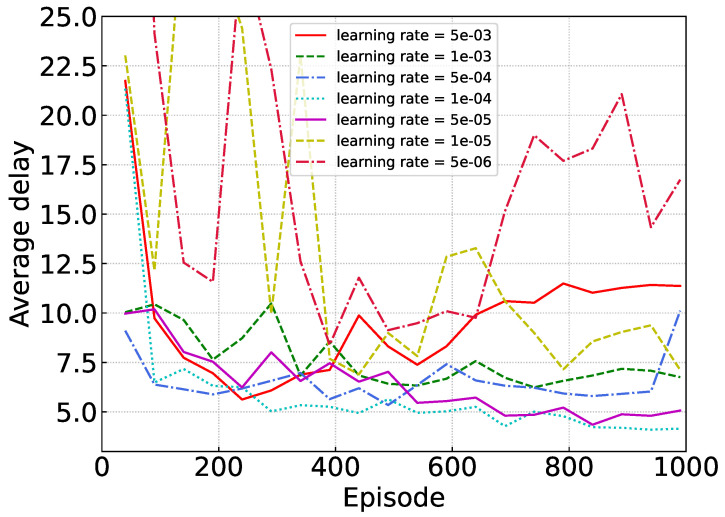
Convergence under different learning rates.

Figure 4 illustrates the convergence of our proposed algorithm with varying learning rates. If the learning rate is excessively high, for instance, 5 × $10^{-3}$, the neural network fails to converge, as the parameters oscillate beyond the acceptable range. Conversely, a learning rate that is excessively low, like 5 × $10^{-6}$, results in the algorithm’s performance hovering around local optima with minimal enhancements.

**Figure 5:**
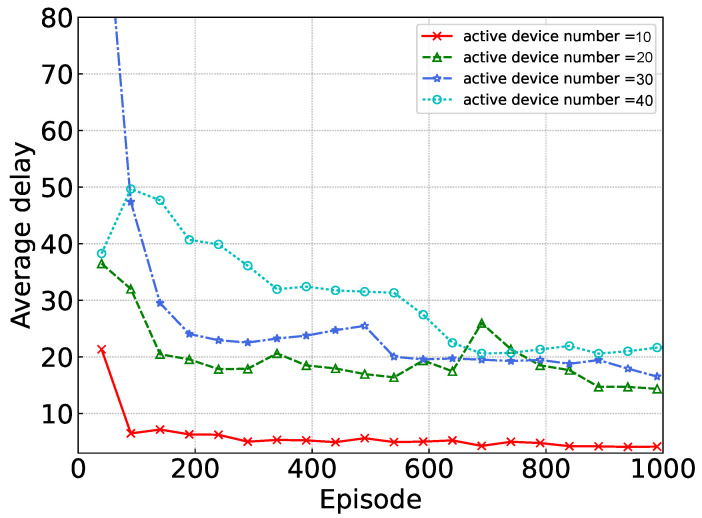
Convergence under different numbers of active devices.

Figure 5 shows the performance of our proposed algorithm with varying numbers of active devices. As the number of active devices increases, the tasks generated per time slot also rise, consequently elevating the system load when the edge server and idle devices remain constant. This increase in system load contributes to a rise in average delay. Nonetheless, our proposed algorithm converges even in the face of these escalating challenges within the environment.

**Figure 6:**
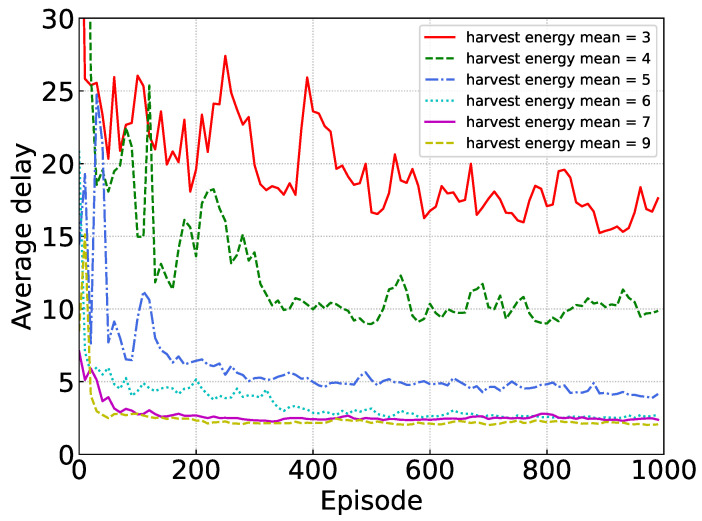
Performance under different harvested energy means.

Figure 6 shows the performance of our proposed algorithm under various mean values of harvested energy. A higher mean harvested energy value indicates a less stringent energy constraint. From Figure 6, it can be seen that our algorithm shows good convergence when the mean value is greater than 5. Moreover, as the mean value increases, the algorithm converges faster. Meanwhile, we can observe that when the average harvested energy surpasses 6 units, the captured energy proves adequate to fulfill the device’s requirements, with no additional benefit from further increases. Conversely, an insufficiency in harvested energy leads to temporary operational interruptions.

**Figure 7:**
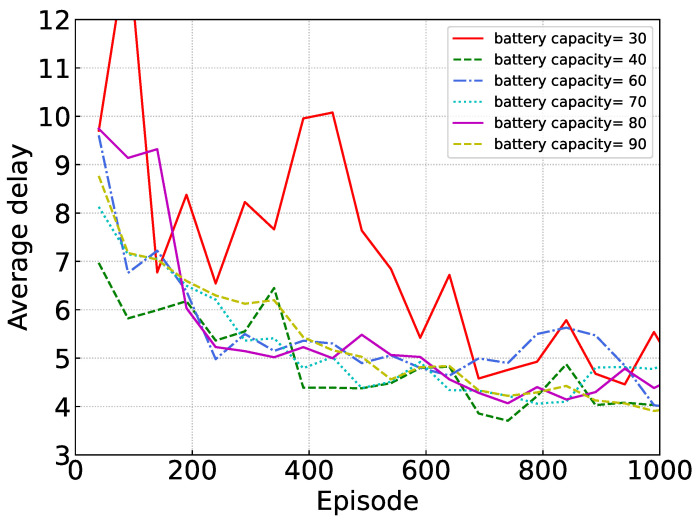
Performance under different battery capacities.

Figure 7 shows the performance of our proposed algorithm under different battery capacities. It is evident that suboptimal outcomes are only evident when the battery capacity is limited, as seen with a value like 30. Conversely, when the battery capacity is substantial, the algorithm consistently delivers comparable performance, regardless of the specific capacity, underscoring its efficient utilization of the captured energy.

**Figure 8:**
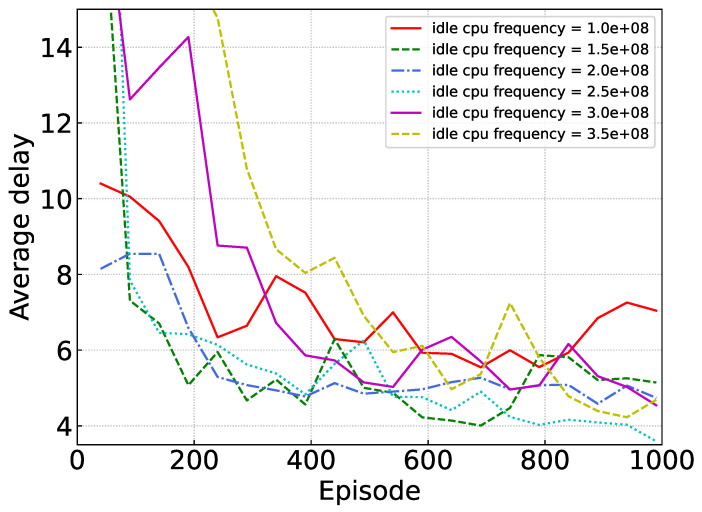
Performance under different idle MD CPU frequencies.

Figure 8 shows the performance of our proposed algorithm under varying CPU frequencies of idle devices. As the CPU frequency of idle devices increases, indicating a stronger processing capability, tasks offloaded to the device are completed more swiftly. However, due to stringent energy constraints on idle devices, higher CPU frequencies result in increased energy consumption. The harvested energy from the battery may prove insufficient to meet the energy demands of high CPU frequencies, leading to a rise in average delay rather than a reduction. It is evident from Figure 8 that the average delay is minimized when the CPU frequency is 2.5 × $10^{8}$.

## 7. Conclusions

In this paper, we introduced a task-offloading framework that leverages energy harvesting and DVFS techniques in D2D-assisted MEC networks to minimize task delay within energy constraints. To tackle limited information exchange stemming from communication channels and user privacy concerns, we developed an MAPPO-based algorithm called MACO. MACO follows a centralized training with decentralized execution framework to determine offload targets and CPU frequencies. Our experimental results showcased the efficacy and resilience of our proposed algorithm. For future research, we aim to introduce a radio frequency-based charging method to enhance device battery life and enhance overall system controllability.

## Figures and Tables

**Table 1:** Notation definitions.

Symbol	Definition
D	The set of mobile deivces
M	The set of active devices
N	The set of idle devices
A(t)	The joint offloading decision at time slot *t*
F(t)	The joint CPU frequency selection at time slot *t*
F	Optional CPU frequency collection
$a_{m}^{t}\left(t\right)$	The offloading decision of the *m*th active device at time slot *t*
$f_{m}$(t)	The CPU frequency selection of the *m*th active device at time slot *t*
$w_{m}^{t}$	The task generated by active device *m* at time slot *t*
$s_{m}^{t}$	The size of task $w_{m}^{t}$
$c_{m}^{t}$	The CPU cycles required to execute a bit of task $w_{m}^{t}$
$Q_{d}^{comp}$	The computing queue of mobile device *d*
$Q_{m}^{tran}$	The transmission queue of active device *m*
$l_{m,d}^{comp}\left(t\right)$	The time slot when task w(m,t) is fully processed at device *d*
$l_{m}^{tran}\left(t\right)$	The time slot when task w(m,t) transmission is completed at device *m*
$r_{m}\left(t\right)$	The transmission rate of mobile device *m* at time slot *t*
$p_{m}$	The transmission power at device *m*
$h_{m}^{d}\left(t\right)$	The channel gain between active device *m* and idle device *d* at slot *t*
$N_{0}$	The white noise
$B_{m}\left(t\right)$	The bandwidth of device *m* at time slot *t*
L(m,t)	The total duration of task w(m,t) from generation to execution completion
$RB_{d}\left(t\right)$	Battery energy of device *d* in time slot *t*
$e_{d}^{c}\left(t\right)$	Energy consumed by device *d* in time slot *t*
$e_{d}^{h}\left(t\right)$	Energy harvested by device *d* in time slot *t*
$S_{m}\left(t\right)$	Local observation information of device *m* on time slot *t*
S(t)	Global state information on time slot *t*
r(t)	Reward in time slot *t*

**Table 2:** The system parameter settings.

Parameter	Value
Active device number |M|	10
Idle device number *N*	4
Idle CPU frequency $f^{idle}$	$2\times 10^{8}$ cycles/s
Edge server CPU frequency $f_{0}$	$4\times 10^{8}$ cycles/s
CPU power parameter δ	$10^{-27}\;\mathrm{Watt}\cdot s^{3}/{\mathrm{cycle}}^{3}$
Active device optional CPU frequency $f_{d}$	$\left[2\times 10^{8},2.5\times 10^{8},3\times 10^{8}\right]$ cycles/s
Total bandwidth *B*	3 MHz
White noise $N_{0}$	$10^{-3}$ Watt
Task generation probability	0.5
Minimum task size $s_{min}$	$2\times 10^{5}$ bits
Maximum task size $s_{max}$	$5\times 10^{5}$ bits
Minimum task complexity $c_{min}$	500 cycles/bit
Maximum task complexity $c_{min}$	1000 cycles/bit
Device transmission power $p_{d}$	5 Watt
Maximum battery capacity $RB^{max}$	50 J

## Data Availability

Available on request.
